# Cryptotanshinone Reverses Reproductive and Metabolic Disturbances in PCOS Model Rats via Regulating the Expression of CYP17 and AR

**DOI:** 10.1155/2014/670743

**Published:** 2014-04-03

**Authors:** Jin Yu, Dongxia Zhai, Li Hao, Danying Zhang, Lingling Bai, Zailong Cai, Chaoqin Yu

**Affiliations:** ^1^Department of Traditional Chinese Medicine, Changhai Hospital, Second Military Medical University, 168 Changhai Road, Shanghai 200433, China; ^2^Traditional Chinese Medicine University of Shanghai, Shanghai 201203, China; ^3^Basic Medical College, Henan College of Traditional Chinese Medicine, Zhengzhou 450008, China; ^4^Department of Biochemistry and Molecular Biology, 800 Xiangyin Road, Second Military Medical University, Shanghai 200433, China

## Abstract

*Objective*. To explore the effect of Cryptotanshinone on reversing the reproductive and metabolic disturbances in polycystic ovary syndrome (PCOS) model rats and the possible regulatory mechanisms. *Methods*. PCOS model rats were induced by subcutaneous injection of dehydroepiandrosterone (DHEA) and verified by histological screening of vaginal exfoliated cells. After Cryptotanshinone intervention, the rats' body weight and ovary morphological were observed; the serum biochemical assessments were analyzed by radioimmunoassay (RIA) and key genes and proteins related with anabolism of androgen and insulin were detected by Real-Time PCR and Immunohistochemical (IHC). *Results*. The estrous cyclicity of PCOS model rats was significantly recovered by Cryptotanshinone. The body weight, ovarian coefficient, and ovarian morphology had been improved and the serum biochemical indicators including testosterone (T), androstenedione (A2), luteinizing hormone (LH), LH/follicle stimulating hormone (FSH), sexual binding globulin (SHBG), low density cholesterol (LDL-C), fasting insulin (FINS) were reversed after Cryptotanshinone intervention. Specifically, the levels of Cytochrome P450, 17-a hydroxylase/17,20 lyase (CYP17), and androgen receptor (AR) were downregulated significantly. *Conclusions*. Our data suggest that Cryptotanshinone could rebalance reproductive and metabolic disturbances in PCOS model rats and could be a potential therapeutic agent for the treatment of PCOS.

## 1. Introduction

Polycystic ovary syndrome (PCOS), a complex genetic condition, is a highly prevalent heterogeneous syndrome of clinical and/or biochemical hyperandrogenism, oligoanovulation, and polycystic ovaries (PCO), excluding other endocrinopathies according to the 2003 Rotterdam criteria [1, 2]. Women with PCOS are at increased risk of reproductive abnormalities, and two-thirds of them also have metabolic dysfunction and, thereby, have an increased risk of developing type 2 diabetes mellitus (T2DM) and cardiovascular disease (CVD) [3]. So the therapy of reversing the high level of androgen and improving the insulin sensitivity is significant for PCOS patients.

Cryptotanshinone, traditionally known as tanshinone, was originally isolated from the dried roots of* Salvia miltiorrhiza* Bunge [4]. In traditional Chinese medicine, Cryptotanshinone has been widely prescribed for several pathologies, including acne, cardiovascular, and some endocrine metabolic diseases such as diabetes [5]. Our study made efforts to explore the effects and mechanisms whereby Cryptotanshinone ameliorates androgen excess and insulin resistance in a PCOS rat model induced by DHEA.

## 2. Materials and Methods

### 2.1. Animals

SPF grade female Wistar rats (age 21 d; body wt 35–40 g; *n* = 50) were purchased from Shanghai Slack laboratory animal co., LTD (license: SCXK(HU)2007-0003) and raised in Second Military Medical University Animal Center, 25°C constant temperature (Humidity 50%), 12 h light : 12 h dark cyclical alternates. All procedures described here were reviewed and approved by the Ethical Committee of Second Military Medical University.

### 2.2. Drugs

Cryptotanshinone (purity 99%) was purchased from Shanghai Ziyi biological technology co., LTD. (batch number: E - 0024); DHEA (purity ≥ 99%) was purchased from Sigma (batch number: D4000); Sesame oil (for injection) was purchased from Sigma (batch number: S3547).

### 2.3. Rat Model

The PCOS rat model, according to Anderson et al. [6, 7], was induced by subcutaneous injection of DHEA and verified by histological screening of vaginal exfoliated cells. Specifically, on the age of 23 d, 40 rats (as the DHEA-ed group) were injected subcutaneously with DHEA 6 mg/kg/day (dissolved into 0.2 mL sesame oil), while the other 10 (as the oil-ed group) were injected subcutaneously with sesame oil 0.2 mL/day. Both of the two groups were administrated for 20 days; then the test of vaginal exfoliated cytology was taken for 10 continuously days and the successful PCOS model rats were selected. The oil-ed group rats have the normal estrous cycle.

### 2.4. Drug Intervention

At age 53 d, 24 successful PCOS model rats chosen from the DHEA-ed group, randomly, were divided into two groups: one was Model group (*n* = 12) and the other was Drug group (*n* = 12). The 10 normal rats (the oil-ed group ones) were referred to as Control group (*n* = 10); both Model group rats and Control group rats were arranged to orally receive the following vehicle: normal saline (dose: 0.01 mL/body wt(g)/day), while Drug group rats orally received Cryptotanshinone (dissolved in normal saline, dose: 0.027 mg/body wt(g)/day) [8]. All rats of the three groups were treated for 4 weeks between 9:00 and 10:00 A.M everyday.

### 2.5. RIA Analysis

Biochemical assessments of rat serum weredetected by RIA analysis. The abdominal aortic blood (about 4 mL) was obtained from the experimental rats anaesthetized by 3.5% chloral hydrate (350 mg/kg body mass). After being left for 2 h at room temperature, the whole blood was centrifuged for 15 min (2500 g) and the serum was separated and stored in −20°C refrigerator before test. At last, the serum levels of T, A2, estradiol (E2), LH, FSH, SHBG, triglycerides (TG), total cholesterol (TC), high density cholesterol (HLD-C), LDL-C, fasting plasma glucose (FPG), and FINS were detected by RIA kits. This part of the test experiment was coperformed with Shanghai Audi kang Biotechnology co., LTD. China.

### 2.6. Light Microscope Analysis

Bilateral ovarian tissues of rats were surgically detached and the wet weight values were obtained from electronic scales. One side of the ovarian tissues was immediately stored in liquid nitrogen; the other side was quickly fixed in 10% Formalin for 24 h and then dehydrated in increasing concentrations of ethanol, followed by immersion in xylene and embedding in paraffin wax. The paraffin-embedded ovary sections (4 *μ*m) were stained with hematoxylin and eosin and analyzed under a conventional light microscope. In addition, 3 fields of each ovary section were selected randomly and the number of follicles (including follicles at all levels) was counted. Two investigators, blinded to the sections' origin, independently analyzed the sections under a conventional microscope, took the available pictures, and calculated the number of follicles.

### 2.7. Real-Time PCR Analysis

Six ovarian tissues from each group preserved in liquid nitrogen were randomly selected for Real-Time PCR analysis. Total RNA was isolated from ovarian tissues using Trizol reagent and following the manufacturer's instructions. Total RNA (3 *μ*g) was reverse transcribed with Reverse Transcription kit (Tatar, USA) as described by the manufacturer. The resulting cDNA was diluted 10-fold in sterile water and aliquots were subjected to Real-Time PCR. PCR primer pairs for the analysis were designed and synthetized by Invitrogen biological technology co., LTD, Shanghai, China (Table 1). Finally, the 3*β*-hsd, cyp17, cyp19, ar, igf-1, and gdf-9 mRNA levels were, respectively, analyzed by quantitative PCR instrument (ROTORGENE6000, Carbett.). The relative expression of each target gene compared to *β*-actin was calculated using the 2^ΔΔCt^ method.

### 2.8. IHC Analysis

Immunohistochemistry method was performed using a two-step EnVision/HRP technique (Dako Cytomation, Denmark) according to the manufacturer's instruction as the description of protocol referenced to the publication [9]. The proteins included 3*β*-hydroxy steroid dehydrogenase (3*β*-HSD), CYP17, Cytochrome P450 aromatase (CYP19), AR, insulin-like growth factors (IGF-1), and growth differentiation factor-9 (GDF-9).  Table 2 lists the antibodies used in this research. Two investigators assessed degree of immunostaining by blinded examination (Table 2).

### 2.9. Statistical Analysis

Statistical evaluations were analyzed using the Statistical Package for the Social Sciences (SPSS version 16.0). Values are expressed as means ± SD. Independent *t*-tests assessed differences and the one-way ANOVA was used to analyze the differences among groups. *P* < 0.05 was considered to be statistically significant.

## 3. Results

### 3.1. Estrous Cyclicity

According to the predominant cell type in vaginal smears obtained daily (10 consecutive days) determined by microscopic analysis, the estrous cyclicity of rats can be observed. After being injected with DHEA for 20 days, the estrous cyclicity of 25 rats was in disorder in DHEA-ed group (40 rats in all), and the success rate of PCOS rat model induced by DHEA was 62.50%. In addition, the disordered estrous cycle of 7 rats in Drug group (12 rats in all) was reversed after Cryptotanshinone intervention for 28 days and the recovery rate was 58.33% which was statistically significant comparing with the natural recovery rate of Model group 8.33% (Tables 3 and 4).

### 3.2. Body Weight and Ovaries Quotiety

**Figure 1:**
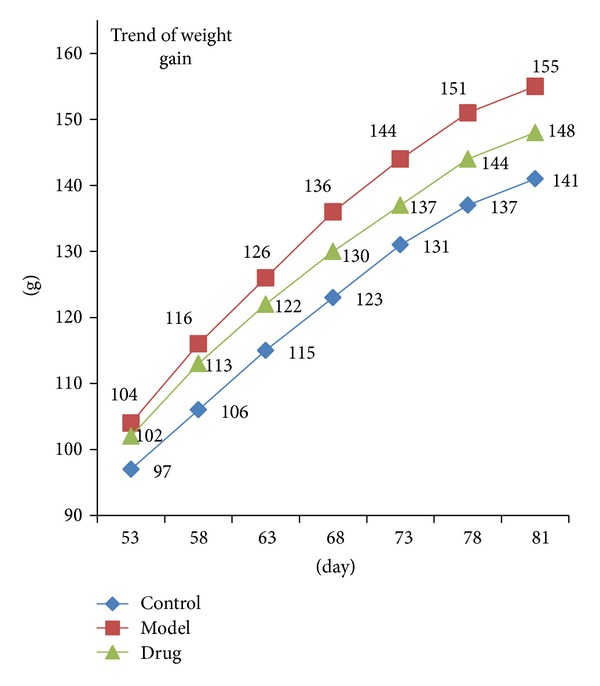
Trends of the weight gain in three groups. The blue, red, and green lines represent the Control group, Model group, and Drug group, respectively. As is shown above, a gain in weight value of Drug group had been slowed down compared to the Model group rats.

From the age of 53 d, the rats of each group were weighed as a frequency of once in five daysuntil the end of the drug intervention; the average weight value was calculated and the trend of the weight gains was observed (Figure 1).

**Figure 2:**
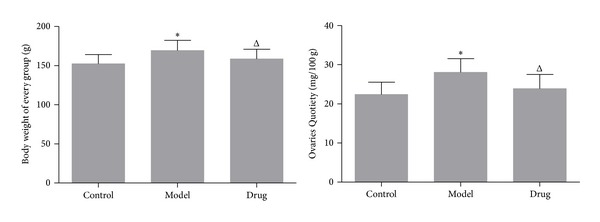
The comparison of body weight value and Ovaries Quotiety among the three groups; Model group (*n* = 12) versus Control group (*n* = 10):  *P < 0.05; Drug group (*n* = 12) versus Model group (*n* = 12): ^Δ^ *P* < 0.05.

At the age of 91 d, all rats were fasting but water for 12 h and then weighed by electronic scale and compared between groups. The average body weight value of Model group rats (169.22 ± 13.25 g) was significantly higher than that of both the Control group rats (152.36 ± 11.84 g) and the Drug group rats (158.57 ± 12.47 g). In addition, Ovaries Quotiety = ovarian weight/body weight (mg/100 g). According to the results of statistical data, the average Ovaries Quotiety of Model group rats [(28.08 ± 3.48 mg/100 g)] was significantly increased compared with the Control group ones [(22.40 ± 3.15 mg/100 g)]; while compared with the Model group, the average Ovaries Quotiety of the Drug group was statistically decreased [(23.90 ± 3.61 mg/100 g)] (Figure 2).

### 3.3. Biochemical Assessments

After detection by RIA, there were significant differences in serum level of T, A2, E2, LH, LH/FSH, SHBG, TC, LDH-C, HDL-C, and FINS between Control group and Model group (*P* < 0.05). To compare Model group with Drug group, there were obvious differences in serum concentration of T, A2, LH, LH/FSH, SHBG, LDH-C, and FINS, while the serum levels of E2, TC, and HDL-C were nonsignificant between the two groups. What is more, there were no statistical differences in serum level of FSH, TG, and FPG among the three groups (Table 5).

### 3.4. Pathological Morphology

**Figure 3:**
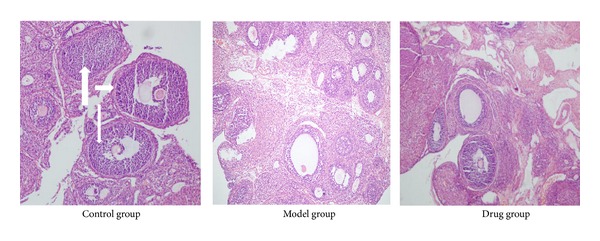
Pathological morphology of ovarian tissues analyzed by light microscope. As is shown above, there were significant differences in color, the numbers of follicles (short thick arrows) and corpora lutea (long thick arrow), and the numbers of granulosa cell layer (long thin arrows) not only between the Model group and the Control group but also between the Drug group and the Model group.

Light microscope analysis showed no structural abnormalities in Control group rats: the ovarian tissue was pink, follicles (the number was 5 ± 2/field) and corpora lutea were in varying stages of development, and granulosa cell layers were normal (the number of granulosa cell layer was 6–8). Differences in Model group rats, however, were significant: the color of the ovarian tissue was lighter as an overall observation; the number of cystic follicles (a large fluid-filled cyst, the number was 15 ± 4/field) increased and the granulosa cell layers were abnormal (the number. of granulosa cell layer was 2–4 or even none); besides, the number of corpora lutea dropped sharply. After Cryptotanshinone intervention, the ovarian pathological morphology of Drug group was greatly reversed, the color of the ovarian tissue and the number of granulosa cell layer had a certain degree of recovery, and the number of cystic follicles (the number was 8 ± 3/field) decreased while the number of corpus luteums increased (Figure 3).

### 3.5. Genes and Proteins Expression

**Figure 4:**
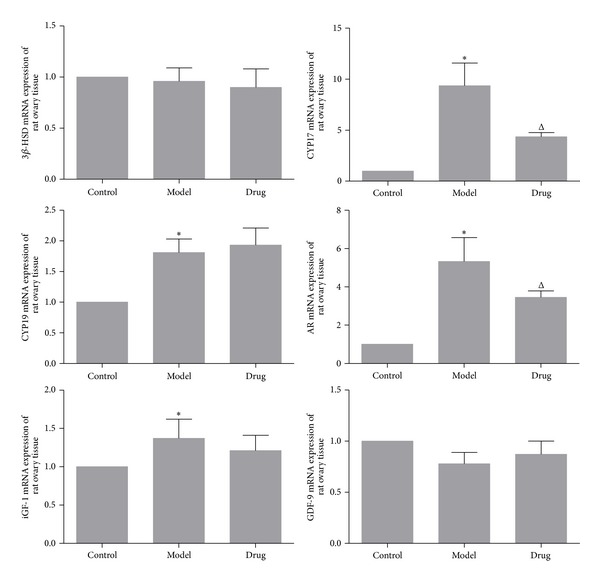
The genes expression of ovarian tissues analyzed by Real-Time PCR. As is shown above, there were no obvious differences between 3*β*-hsd mRNA and gdf-9 mRNA among the three groups. The expression of cyp17 mRNA (9.35 ± 2.23), cyp19 mRNA (1.81 ± 0.22), ar mRNA (5.33 ± 1.24), and igf-1 mRNA (1.37 ± 0.25) in Model group was increased significantly than in the Control group (1.00 ± 0.00). After drug intervention, the expression of cyp17 mRNA (4.36 ± 0.39) and ar mRNA (3.45 ± 0.34) was statistically decreased, while cyp19 mRNA and igf-1 mRNA had no statistical differences (cyp19 mRNA: 1.93 ± 0.28; igf-1 mRNA: 1.21 ± 0.20). Model group (*n* = 6) versus Control group (*n* = 6): *P < 0.05; Drug group (*n* = 6) versus Model group (*n* = 6): ^Δ^ *P* < 0.05.

**Figure 5:**
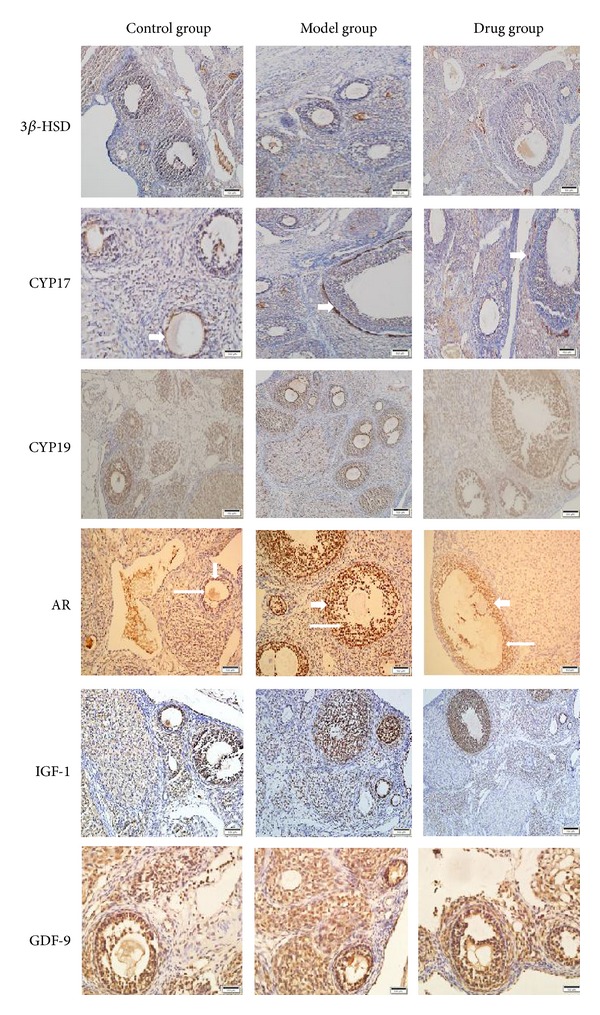
Immunohistochemical staining of 3*β*-HSD, CYP17, CYP19, AR, IGF-1, and GDF-9 in three groups. As is shown above, CYP17 was primarily localized in theca cells (short thick arrows), AR was primarily expressed both in theca cells (short thick arrows) and in granulosa cells (long thin arrows), while the expressions of other proteins were not obvious.

**Figure 6:**
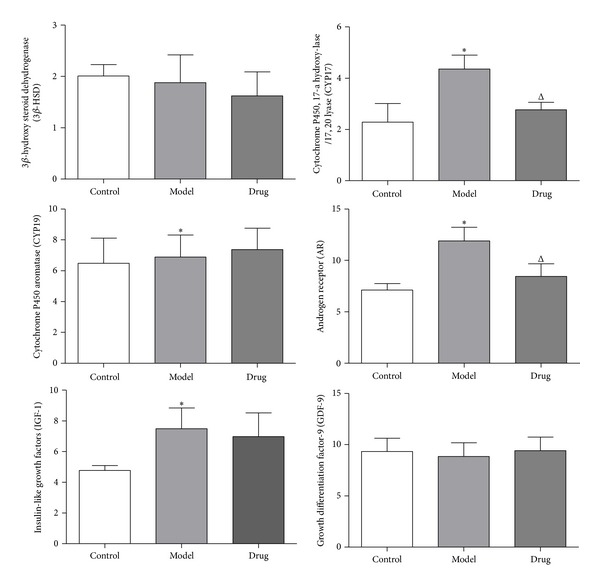
The protein expression of ovarian tissues analyzed by IHC. As is shown in the histogram above, except 3*β*-HSD and GDF-9, the expression of CYP17, CYP19, AR, and IGF-1 significantly increased in Model group than that of Control group, while in Drug group only CYP17 and AR were decreased according to statistics. Model group (*n* = 6) versus Control group (*n* = 6): *P < 0.05; Drug group versus Model group (*n* = 6): ^Δ^ *P* < 0.05.

The levels of key genes and proteins related to anabolism of androgen and insulin were detected by Real-Time PCR and IHC. The expression of cyp17, cyp19, ar, and igf-1 in Model group was significantly higher than that of the Control group; after Cryptotanshinone intervention, the expression of cyp17 and ar was reversed; however, the level of cyp19 and igf-1 was nonsignificant. The level of 3*β*-hsd and gdf-9 had no statistical differences according to the analysis of Real-Time PCR. Besides, as IHC analyzed, except the fact that the protein CYP17 and AR had been downregulated significantly, it seemed that Cryptotanshinone had little effect on reversing the expression of protein CYP19 and IGF-1 increased in Model group, while the protein 3*β*-HSD and GDF-9 had almost no significant differences among groups, all of which were consistent with the results of Real-Time PCR (Figures 4, 5, and 6).

## 4. Discussion

**Figure 7:**
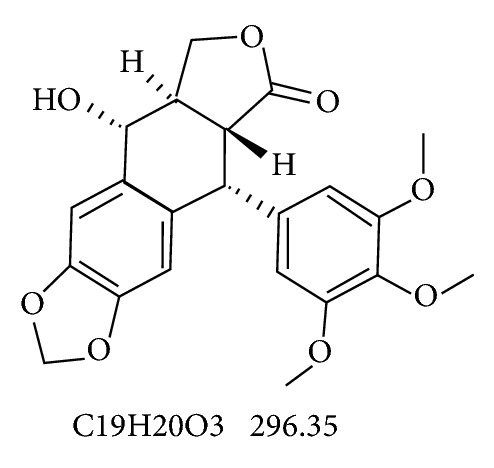


Cryptotanshinone, a kind of fat-soluble 2 terpenoids material [10], has been isolated from the herb of* Salvia miltiorrhiza* and identified as the major chemical constituent [11]. Its molecular formula is C_19_H_20_O_3_, the Mol. wt. is 296.35, and the chemical structural is as in Figure 7.

Modern pharmacological studies have shown that Cryptotanshinone has the effects of antibacterial, anti-inflammatory, antiobesity, and antidiabetic activity [12, 13] and has been prescribed for several diseases, including coronary heart disease (CHD), angina pectoris, and myocardial damage. Recently, some scholars have focused their interests on researching the relationship between Cryptotanshinone and some endocrine metabolic diseases. For instance, Yang et al. [8] reported that Cryptotanshinone could reverse the disturbances in prenatally androgenized rats; Qi et al. [14] discovered that Cryptotanshinone could improve insulin resistance of pig ovarian theca cell cultured in vitro.

In our experiment, we successfully induced the PCOS rat model by subcutaneous injection DHEA, a kind of exogenous androgen. The rat model, in general, recapitulated the reproductive and metabolic features of human PCOS, including the irregular estrous cycles, increased body weight, and ovarian coefficient and the abnormal serum biochemical level of T, A2, E2, LH, LH/FSH, SHBG, TC, LDH-C, HDL-C, and FINS. After Cryptotanshinone intervention, the estrous cyclicity of model rats was obviously restored. Besides, the body weight, ovarian coefficient, and ovarian morphology of PCOS model rats had been improved. Even more noteworthy is that the serum biochemical indicators including T, A2, LH, LH/FSH, SHBG, LDH-C, and FINS had been reversed significantly.

Ovary is one of the sources of androgen and insulin anabolic in the human body. In PCOS ovarian tissue, theca cells and granule cells abnormally express mRNA for key genes involved in anabolic of androgen and insulin, including 3*β*-hsd, cyp17, cyp19, ar, igf-1, and gdp-9. Our data showed that the levels of cyp17 and ar were obviously downregulated by Cryptotanshinone; accordingly, the protein levels of these two genes were also downregulated as detected by IHC.

The possible regulatory mechanisms of Cryptotanshinone on reversing the reproductive and metabolic disturbances in PCOS model rats might be as follows: large amount of exogenous DHEA prompts the pituitary gland sensitive to gonadotrophin-releasing hormone (GnRH) and increases the excessive secretion of LH; the increased GnRH/LH can enhance the activity of cytochrome P450 and 17-*α* hydroxylase/17,20 lyase (coded by gene cyp17), one of the key enzymes in theca cells, and result in the synthetic of A2 and T increasing [15]. The excessive androgen in ovarian, on one hand, inhibits the development of follicle and makes it difficult to form a dominant follicle and at last leads to the state of polycystic ovary sample (PCO) [16]; On the other hand, small follicles in the ovaries can secrete E2, the equivalent of the early follicles, and A2 can be converted into estrone (E1) by cytochrome P450 aromatase (coded by gene cyp19) outside the tissues. Sustained release of E1 and a certain level of E2 effect on the hypothalamus and pituitary [17] make a positive feedback on LH secretion, further increase the amplitude and frequency of LH secretion, and make the ratio of LH/FSH inversed. Cryptotanshinone can directly and/or indirectly reduce the levels of A2 and T probably by inhibiting the expression CYP17 and reducing the biosynthesis of androgen. In addition, our test also found that the level of AR was downregulated by Cryptotanshinone, thereby reducing the role of androgen by limiting the expression of AR.

Modern scientific researches widely believe that PCOS is a kind of metabolic disorder; most of PCOS patients suffer from insulin resistance and lipid metabolism dysfunction. Hyperinsulinemia of PCOS could directly impact the hypothalamus or pituitary gland and raise the level of LH and therefore indirectly enhance the secretion of androgen mediated by LH [18]. At the same time, hyperinsulinemia could suppress the synthesis of SHBG in liver and cause the increasing of free testosterone (FT) [19]. At present, it is believed that the level of SHBG in peripheral blood can reflect the degree of insulin resistance [18]. In our research, we found the serum biochemical indices of SHBG and FINS significantly regulated after Cryptotanshinone intervention. Besides, the level of igf-1 had a downward trend in Cryptotanshinone group, and although it had no statistical significance, we cannot exclude the situation that Cryptotanshinone enhanced insulin sensitivity and improved insulin resistance by inhibiting the expression of IGF-1. In addition, some reports showed that androgen can strengthen lipoprotein lipase (LPL) catabolism [20] and insulin has an upregulated effect on LDL-C receptor activity [21]. Interestingly, the serum biochemical level of LDL-C was also descended after Cryptotanshinone intervention in our research.

## 5. Conclusions

Cryptotanshinone can reverse the reproductive and metabolic disturbances in PCOS model rats via downregulating the expression of CYP17 and AR, and it may indirectly increase the insulin sensitivity. Therefore, it could be a potential therapeutic agent for the treatment of PCOS patients.

## Figures and Tables

**Table 1:** The primer sequences of target genes.

Target gene	Primer sequences
Rat *β*-actin	F5′-CACAATGCTGGGACACAAAC-3′
	R5′-TGGCGTGAGCAGTTTATCAG-3′

Rat 3*β*-hsd	F5′-GTGTGCCAGCCTTCATCTAC-3′
	R5′-GGAGCAGTGGTGATGTATGG-3′

Rat cyp17	F5′-ATCAGGCCGGTGGCTCCCAT-3′
	R5′-TCGGGGACCAGCTCCGAAGG-3′

Rat cyp19	F5′-CCATCTGGTCTCCTGCTAG-3′
	R5′-CCACTTACCCTCAACACACA-3′

Rat ar	F5′-CGTCGCTCCTGGGAGGTCCA-3′
	R5′-CTGCTGCCAGAGCAGCCCAG-3′

Rat igf-1	F5′-ACATCTCTTCTACCTGGCACTCT-3′
	R5′-AAGCAACACTCATCCACAATG-3′

Rat gdf-9	F5′-AGCTCAAATGGGACAAACTGGAT-3′
	R5′-GGGACAGTCCCCTTTACACTACCT-3′

**Table 2:** Antibodies used in this research.

Antibody	Host species	Source
3*β*-HSD	Rabbit	Santa Cruz
CYP17	Goat	Santa Cruz
CYP19	Rabbit	Santa Cruz
AR	Rabbit	Santa Cruz
IGF-1	Rabbit	Santa Cruz
GDF-9	Goat	Santa Cruz
Goat anti-rabbit IgG, Rabbit anti-goat IgG		Santa Cruz

**Table 3:** The success rate of PCOS rat model induced by DHEA.

Method	Total number	Disorder number	Success rate
Subcutaneous injection of sesame oil	10	0	0.00%
Subcutaneous injection of DHEA	40	25	62.50%

**Table 4:** The recovery rate of Cryptotanshinone intervention.

Group	Total number	Recovery number	Recovery rate
Model group	12	1	8.33%
Drug group	12	7	58.33%*

Drug group versus Model group: *P < 0.05.

**Table 5:** Serum biochemical assessments by radioimmunoassay.

Radioimmunoassay index		T	A2	E2	LH	FSH	LH/FSH	SHBG	TC	TG	LDH-C	HDL-C	FPG	FINS
Group	Number	(nmol/L )	(nmol/L)	(pmol/L)	(IU/L)	(IU/L)	(IU/L)	(nmol/L)	(nmol/L)	(nmol/L)	(*μ*mol/L)	(*μ*mol/L)	(mIU/L)	(mIU/L)
Control group	10	0.76 ± 0.07	9.24 ± 2.34	88.76 ± 5.23	28.21 ± 3.82	12.42 ± 1.92	2.27 ± 0.13	40.52 ± 3.76	7.22 ± 1.37	1.00 ± 0.22	32.04 ± 7.56	81.68 ± 15.17	5.36 ± 0.09	5.57 ± 1.01

Model group	12	1.03 ± 0.14*	16.02 ± 6.31*	126.73 ± 17.08*	32.82 ± 1.22*	10.36 ± 2.25	3.17 ± 0.31*	28.52 ± 5.37*	8.63 ± 2.50*	1.89 ± 0.34	55.95 ± 9.85*	140.50 ± 19.91*	6.19 ± 0.17	13.79 ± 3.74*

Drug group	12	0.90 ± 0.11^△^	12.31 ± 2.38^△^	121.46 ± 9.56	29.04 ± 5.11^△^	10.57 ± 2.41	2.75 ± 0.18^△^	35.64 ± 4.82^△^	7.68 ± 1.00	2.05 ± 0.44	49.89 ± 7.18^△^	162.32 ± 30.51	6.25 ± 0.21	9.95 ± 1.68^△^

Model group versus Control group: *P < 0.05; Drug group versus Model group: ^△^ *P* < 0.05.
